# A Variable Height Microfluidic Device for Multiplexed Immunoassay Analysis of Traumatic Brain Injury Biomarkers

**DOI:** 10.3390/bios11090320

**Published:** 2021-09-07

**Authors:** Alyse D. Krausz, Frederick K. Korley, Mark A. Burns

**Affiliations:** 1 Biomedical Engineering Department, University of Michigan, Ann Arbor, MI 48109, USA; 2 Department of Emergency Medicine and Michigan Medicle, University of Michigan, Ann Arbor, MI 48109, USA; korley@umich.edu; 3 Chemical Engineering Department, University of Michigan, Ann Arbor, MI 48109, USA

**Keywords:** microfluidics, traumatic brain injury, multiplex, immunoassay, biomarkers

## Abstract

Traumatic brain injury (TBI) is a leading cause of global morbidity and mortality, partially due to the lack of sensitive diagnostic methods and efficacious therapies. Panels of protein biomarkers have been proposed as a way of diagnosing and monitoring TBI. To measure multiple TBI biomarkers simultaneously, we present a variable height microfluidic device consisting of a single channel that varies in height between the inlet and outlet and can passively multiplex bead-based immunoassays by trapping assay beads at the point where their diameter matches the channel height. We developed bead-based quantum dot-linked immunosorbent assays (QLISAs) for interleukin-6 (IL-6), glial fibrillary acidic protein (GFAP), and interleukin-8 (IL-8) using Dynabeads^TM^ M-450, M-270, and MyOne^TM^, respectively. The IL-6 and GFAP QLISAs were successfully multiplexed using a variable height channel that ranged in height from ~7.6 µm at the inlet to ~2.1 µm at the outlet. The IL-6, GFAP, and IL-8 QLISAs were also multiplexed using a channel that ranged in height from ~6.3 µm at the inlet to ~0.9 µm at the outlet. Our system can keep pace with TBI biomarker discovery and validation, as additional protein biomarkers can be multiplexed simply by adding in antibody-conjugated beads of different diameters.

## 1. Introduction

Traumatic brain injury (TBI) causes significant morbidity and mortality globally. In the United States alone, an estimated 2.87 million people sustain a TBI annually, and 50,000 of these individuals die of their injuries. Furthermore, 2% of the United States population is estimated to have a disability caused by TBI [1,2]. The high rates of morbidity and mortality can be attributed to inadequate diagnostic and treatment methods. No new treatments for TBI have been approved in 30 years, largely due to the heterogeneity of injuries and limited tools for accurate diagnosis and classification of TBI [1,3].

Assessment of TBI injuries is typically done through neurological examination and neuroimaging techniques. While these methods may identify direct tissue damage to the brain, they are more limited in their ability to monitor secondary damage stemming from the initial injury. The primary tissue damage sets off a cascade of secondary injuries, such as neuronal cell death, blood–brain barrier breakdown, edema, and upregulation of inflammatory markers [4,5]. 

Protein biomarkers have been proposed as a way of monitoring the progression of secondary TBI injury and of providing more sensitive diagnostic measures when used in conjunction with imaging and physical examination. Glial fibrillary acidic protein (GFAP) and ubiquitin c-terminal hydrolase L1 (UCH-L1) are currently FDA approved to aid in determining the need for evaluation with a head CT scan [6]. However, TBI biomarker discovery and validation is an ongoing area of research, and multiple candidate molecules and proteins have been proposed [1,4,7,8,9]. It has been suggested that a panel of TBI biomarkers may be necessary to obtain precise diagnostic and prognostic information [10]. Multiplexed immunoassays would facilitate the measurement of multiple TBI biomarkers at once.

Many microfluidic devices have been developed to facilitate multiplexed immunoassays. These devices typically employ surface patterning [11,12,13], color-coded or sequestered microbeads [14,15,16], or large-scale microreactor integration [17] to achieve multiplexing. Because of the complexity of these systems, the microfluidic device often must be redesigned if an additional immunoassay needs to be multiplexed, increasing fabrication and development costs.

To overcome this limitation, we developed a microfluidic system capable of flexible and customizable multiplexing. In this study, we present bead-based quantum dot-linked immunosorbent assays (QLISAs) for GFAP, a marker of astrocyte injury [18]; interleukin-6 (IL-6), a marker of inflammation; and interleukin-8 (IL-8), another marker of inflammation [19,20,21]. GFAP was chosen as a target biomarker since it is currently being incorporated into clinical practice for TBI evaluation [6]. While not TBI specific, IL-6 and IL-8 have been studied in the context of TBI [19,20,21]. IL-6 has recently been associated with neuroimaging findings in patients with mild traumatic brain injury (mTBI), and IL-8 has shown promise as a predictor of persistent fatigue after TBI in children [22,23]. The GFAP, IL-6, and IL-8 QLISAs were analyzed using a variable height microfluidic device fabricated using a modified version of the method originally described by Mena et al. [24]. A different-sized bead was used for each assay, facilitating the passive formation of distinct bands within the variable height device. The QLISAs were decoupled from the variable height channel allowing for the formation of customized multiplexed brain injury barcodes without redesigning the device.

## 2. Materials and Methods

### 2.1. Bead-Based Quantum Dot-Linked Immunosorbent Assay (QLISA) Design

Each bead-based QLISA was developed using the same set of reagent ratios. The volume of capture antibody-coated superparamagnetic beads was chosen such that there was a 5x molar excess of capture antibodies to the maximum protein (either GFAP, IL-6, or IL-8) concentration of interest. The volume of detection antibody was chosen such that there was a 10x molar excess of detection antibodies to the maximum protein concentration of interest. Finally, the volume of quantum dots was chosen such that there was a 2x molar excess of quantum dot to detection antibody.

### 2.2. Glial Fibrillary Acidic Protein (GFAP) QLISA

Human GFAP Capture Antibodies from the Human GFAP Matched Antibody Pair Kit (ab222279, Abcam, Gottingen, Germany) were covalently linked to the surface of superparamagnetic beads (Dynabeads^TM^ M-270 Epoxy, Invitrogen^TM^, Waltham, MA, USA) using the Dynabeads^TM^ Antibody Coupling Kit (Invitrogen^TM^). An amount of 1 mg of superparamagnetic beads suspended in dimethylformamide (DMF, Sigma-Aldrich, St. Louis, MI, USA) was washed with 1 mL of the C1 reagent from the Antibody Coupling Kit. Next, 5 µg (5 µL) of Human GFAP Capture Antibody was added to the beads, followed by 45 µL of C1 reagent and 50 µL of C2 reagent. The mixture was incubated overnight (16–24 h) on a rocker at 37 °C. Following incubation, the antibody-coated beads were washed with 800 µL of HB reagent + 0.1% Tween 20 and 800 µL of LB reagent + 0.1% Tween 20. The antibody-coated beads were then washed three times with 800 µL of SB reagent followed by incubation with 800 µL of SB reagent on a rocker for 15 min at room temperature. The antibody-coated beads were then suspended in 100 µL of SB reagent and stored at 4 °C.

Human GFAP Lyophilized Protein from the Human GFAP Matched Antibody Pair Kit (ab222279, Abcam) was reconstituted at 10 ng/µL in distilled water (Gibco^TM^, Shanghai, China). This stock solution was used to spike either normal human serum (S1-100ML, Sigma-Aldrich) or single donor human whole blood (IWB1K2E10ML, Innovative Research) such that the concentrations were 10,000 pg/mL, 5000 pg/mL, 1000 pg/mL, 500 pg/mL, 100 pg/mL, and 0 pg/mL GFAP. The serum and whole blood were rocked for 15 min before and after spiking with GFAP to ensure a homogeneous sample.

To run the assay, 0.75 µL of antibody-coated beads was placed in a 1.7 mL polypropylene microcentrifuge tube (VWR^®^) along with 50 µL of 1X PBS + 0.1% Tween 20. The tube was then placed on a magnet (DynaMag^TM^-2 Magnet, Invitrogen^TM^), and the supernatant was removed. Next, 100 µL of either spiked human serum or whole blood was added to the beads, and the mixture was incubated at room temperature for 15 min on a rocker. The tube was placed on a magnet again, and the supernatant was removed. An amount of 0.3 µL of biotinylated Human GFAP Detector Antibody from the Human GFAP Matched Antibody Pair Kit, 1 µL of Qdot^TM^ 585 Streptavidin Conjugate (Invitrogen^TM^), and 50 µL of 1X PBS + 0.1% Tween 20 was then added to the beads, and the mixture was incubated at room temperature for 15 min on a rocker. The beads were then washed three times in 1X PBS + 0.1% bovine serum albumin (BSA) (A7888, Sigma-Aldrich), washed once in 1X PBS, and resuspended in either 100 µL of 1X PBS or 100 µL of StartingBlock^TM^ (PBS) (Shanghai, China) Blocking Buffer (Thermo Scientific^TM^ (Shanghai, China)).

### 2.3. Measurement of GFAP in Clinical Serum Samples

Ten serum samples from emergency department patients who were evaluated for TBI were tested using the GFAP QLISA in a blind experiment. These serum samples were collected between October 2014 and November 2016, and the GFAP concentration was measured by Quanterix in June 2019. The study was approved by the Institutional Review Boards of the University of Michigan Medical School.

### 2.4. Interleukin-6 (IL-6) QLISA

Human IL-6 Capture Antibodies from the Human IL-6 Matched Antibody Pair Kit (ab246838, Abcam) were covalently linked to the surface of superparamagnetic beads (Dynabeads^TM^ M-450 Epoxy, Invitrogen^TM^) using the Dynabeads^TM^ Antibody Coupling Kit (Invitrogen^TM^). An amount of 200 µL of superparamagnetic beads was washed with 1 mL of the C1 reagent from the Antibody Coupling Kit. Next, 40 µg (40 µL) of Human IL-6 Capture Antibody was added to the beads, followed by 60 µL of C1 reagent and 100 µL of C2 reagent. The mixture was incubated overnight (16–24 h) on a rocker at 37 °C. Following incubation, the antibody-coated beads were washed with 800 µL of HB reagent + 0.1% Tween 20 and 800 µL of LB reagent + 0.1% Tween 20. The antibody-coated beads were then washed three times with 800 µL of SB reagent, followed by incubation with 800 µL of SB reagent on a rocker for 15 min at room temperature. The antibody-coated beads were then suspended in 200 µL of SB reagent and stored at 4 °C.

Human IL-6 Lyophilized Protein from the Human IL-6 Matched Antibody Pair Kit (ab246838, Abcam) was reconstituted at 10 ng/µL in distilled water (Gibco^TM^). This stock solution was used to spike Blocking Buffer (1X PBS + 0.1% BSA + 0.05% Tween 20) such that the concentrations were 25,000 pg/mL, 15,000 pg/mL, 10,000 pg/mL, 5000 pg/mL, 1000 pg/mL, and 0 pg/mL IL-6. All spiked Blocking Buffer samples were inverted to mix.

To run the assay, 0.5 µL of antibody-coated beads was placed in a 1.7 mL polypropylene microcentrifuge tube (VWR^®^) along with 50 µL of 1X PBS + 0.1% Tween 20. The tube was then placed on a magnet (DynaMag^TM^-2 Magnet, Invitrogen^TM^), and the supernatant was removed. Next, 100 µL of spiked Blocking Buffer was added to the beads and the mixture was incubated at room temperature for 15 min on a rocker. The tube was placed on a magnet again, and the supernatant was removed. An amount of 0.72 µL of biotinylated Human IL-6 Detector Antibody from the Human IL-6 Matched Antibody Pair Kit, 2 µL of Qdot^TM^ 525 Streptavidin Conjugate (Invitrogen^TM^), and 50 µL of 1X PBS + 0.1% Tween 20 was then added to the beads, and the mixture was incubated at room temperature for 15 min on a rocker. The beads were then washed three times in 1X PBS + 0.1% BSA (A7888, Sigma-Aldrich), washed once in 1X PBS, and resuspended in either 100 µL of 1X PBS or 100 µL of StartingBlock^TM^ (PBS) Blocking Buffer (Thermo Scientific^TM^).

### 2.5. Interleukin-8 (IL-8) QLISA

Human IL-8 Capture Antibodies from the Human IL-8 Matched Antibody Pair Kit (ab215402, Abcam) were covalently linked to the surface of superparamagnetic beads (Dynabeads^TM^ MyOne^TM^ Tosylactivated, Invitrogen^TM^). An amount of 10 µL of superparamagnetic beads was washed with 1 mL of 0.1 M sodium borate buffer (pH 9.5). Next, 94 µL of 0.1 M sodium borate buffer (pH 9.5), 40 µL of Human IL-8 Capture Antibody, and 66 µL of 3 M ammonium sulfate was added to the beads. The mixture was incubated overnight (16–24 h) on a rocker at 37 °C. Following incubation, the tube was placed on a magnet, and the supernatant was removed. An amount of 200 µL of 1X PBS + 0.5% BSA + 0.05% Tween 20 was then added to the beads, and the mixture was incubated overnight (16–24 h) again at 37 °C. Following the second incubation, the beads were washed three times with 1X PBS + 0.5% BSA + 0.05% Tween 20. The antibody-coated beads were then suspended in 100 µL of 1X PBS + 0.5% BSA + 0.05% Tween 20 and stored at 4 °C.

Human IL-8 Lyophilized Protein from the Human IL-8 Matched Antibody Pair Kit (ab215402, Abcam) was reconstituted at 0.98 ng/µL in distilled water (Gibco^TM^). This stock solution was used to spike Blocking Buffer (1X PBS + 0.1% BSA + 0.05% Tween 20) such that the concentrations were 1000 pg/mL, 800 pg/mL, 500 pg/mL, 200 pg/mL, 100 pg/mL, 10 pg/mL, and 0 pg/mL IL-8. All spiked Blocking Buffer samples were inverted to mix.

To run the assay, 0.5 µL of antibody-coated beads was placed in a 1.7 mL polypropylene microcentrifuge tube (VWR^®^) along with 50 µL of 1X PBS + 0.1% Tween 20. The tube was then placed on a magnet (DynaMag^TM^-2 Magnet, Invitrogen^TM^), and the supernatant was removed. Next, 100 µL of spiked Blocking Buffer was added to the beads and the mixture was incubated at room temperature for 15 min on a rocker. The tube was placed on a magnet again, and the supernatant was removed. An amount of 0.72 µL of biotinylated Human IL-8 Detector Antibody from the Human IL-8 Matched Antibody Pair Kit, 2 µL of Qdot^TM^ 655 Streptavidin Conjugate (Invitrogen^TM^), and 50 µL of 1X PBS + 0.1% Tween 20 was then added to the beads, and the mixture was incubated at room temperature for 15 min on a rocker. The beads were then washed three times in 1X PBS + 0.1% BSA (A7888, Sigma-Aldrich), washed once in 1X PBS, and resuspended in either 100 µL of 1X PBS or 100 µL of StartingBlock^TM^ (PBS) Blocking Buffer (Thermo Scientific^TM^).

### 2.6. Limit of Detection and Coefficient of Variation

The limit of detection (LOD) and coefficient of variation (CV) for each QLISA were calculated as described in Krausz et al. [25]. Briefly, the LOD for each assay was calculated as the value of the blank plus three standard deviations (SD) (*n* = 10). The intra-assay CV was calculated as the SD divided by the mean of replicates (*n* = 3) of the same sample run on the same day. The inter-assay CV was calculated as the average of the CVs for the same sample from three different days.

### 2.7. Variable Height Device Fabrication

The variable height etching procedure presented here is a modified version of the original method described by Mena at al. [24]. MDF polished borosilicate wafers (Wafer Universe) were piranha cleaned. A 200/2000 Å Cr/Au metal mask was evaporated onto the piranha-cleaned wafers with an electron beam evaporator (DV-502A, Denton Vacuum). A 3 µm layer of SPR^TM^220 photoresist was spin-coated using an ACS 200 Cluster Tool (Süss Microtec) and exposed for 15 s with 405 nm at 20 mJ/s using an MA/BA-6 Mask/Bond Aligner (Süss Microtec) and a low-cost photomask (Fineline Imaging). Exposed wafers were developed for 60 s in MF-319 using an ACS 200 Cluster Tool (Süss Microtec). Au and Cr layers were removed by etching for 72 s in TFA gold etchant (Transene) and 16 s in Chromium Etch 1020 (Transene, Danvers, MA, USA).

The masked glass wafers were gradually lowered into 15% hydrofluoric acid (HF) solution (49% *w*/*w* HF diluted with deionized water) using a custom linear screw mechanism. After the channels were etched, the wafer was rinsed in deionized water for 5 min. The resulting etched channels were measured using a surface profilometer (Alpha Step 500, KLA-Tencor). The photoresist was then removed with acetone and isopropyl alcohol, followed by the removal of the Au/Cr layers with their respective etchants. The glass wafer was then dipped in buffered hydrofluoric acid (BHF) for 5 s to remove residual Cr.

Inlet and outlet ports were drilled in a double-side polished p-type silicon wafer that was reversibly bonded to a glass backing wafer using Crystalbond 555 (Ted Pella) heated to 70 °C. Drilling layouts were prepared using Autodesk Fusion 360 and exported as gcode to a CNC router (Tormach PCNC). A peck-drilling operation was used with a 750 µm diamond micro drill (Amplex S-Series 0.030”), a drill speed of 10,000 RPM, a peck height of 50 µm, and a feed rate of 5 mm/min. The drilled silicon wafer and etched glass wafer were anodically bonded on an SB-6E bonder (Süss Microtec) at 350 °C and an applied voltage of 100 V until the current reached 10% of its peak value.

### 2.8. Variable Height Device QLISA Analysis

The sample of processed and resuspended assay beads was placed into a modified 1.7 mL polypropylene microcentrifuge tube (VWR^®^). The microcentrifuge tube was adapted by punching a hole in the lid and a hole in the bottom using a scratch awl and rubber mallet. The pressure input was placed into the hole in the lid (stainless steel dispensing needle with Luer lock connection, McMaster Carr), and the tube (ETFE tubing, 1/16” OD, McMaster Carr) to deliver the sample to the device was placed in the hole in the bottom. The tubing and dispensing needle were secured with two-part epoxy (Gorilla). The tubing was connected to the variable height device channel inlet using a microfluidic probe with a compression sealing mechanism (CorSolutions microfluidic connectors BMP-LP-2X), and pneumatic pressure was applied to drive the sample into the channel that had been primed with either 5% BSA or StartingBlock^TM^ (PBS) Blocking Buffer (Thermo Scientific^TM^). The sample flowed through the channel until a band of beads formed that was visible to the naked eye.

### 2.9. Fluorescent Image Analysis

Fluorescent images (1344 × 1100 pixels) of assay beads trapped in the variable height device were obtained using a Nikon Eclipse Ti inverted microscope and a Retiga R6 monochrome CCD camera (Teledyne Photometrics). A fluorescent image and an epi-illuminated image were obtained for each field of view. ImageJ macros were used to pseudo-flat field correct the epi-illuminated image and to background correct the fluorescent images. A Python script was used to perform the following operations: (1) threshold and binarize the pseudo-flat field-corrected epi-illuminated image to yield B’, (2) perform an elementwise multiplication of B’ and the corresponding background-corrected fluorescent image to yield F’, (3) calculate the sum of all values in B’ to yield b, (4) calculate the sum of all values in F’ to yield f, (5) determine the ratio of f to b to yield RFU/Bead Area. The ImageJ macros and Python script used for image analysis can be found on GitHub (alyseka / assaybead-analysis).

### 2.10. Multiplexed QLISA Analysis

Multiplexed QLISAs were carried out in three different ways. First, the GFAP and IL-6 QLISAs were carried out as described in Section 2.2 and Section 2.4, respectively, using a sample that contained both proteins. The GFAP and IL-6 assay beads were then combined and flowed into the variable height device as described in Section 2.8. Second, the GFAP and IL-6 QLISAs were carried out by combining all assay reagents into one microcentrifuge tube and by using Qdot^TM^ 585 Streptavidin Conjugate as the fluorescent signal for both assays. Once again, a sample was used that contained both proteins. Third, the GFAP, IL-6, and IL-8 QLISAs were carried out as described in Section 2.2, Section 2.4 and Section 2.5, respectively, using a sample that contained all three proteins. The IL-6, GFAP, and IL-8 assay beads were then combined and flowed into the variable height device as described in Section 2.8. All multiplexed QLISA analyses were carried out in spiked buffer samples.

### 2.11. Assay Bead Size Characterization

An amount of 20 µL of either Dynabeads^TM^ M-450 Epoxy, Dynabeads^TM^ M-270 Epoxy, or Dynabeads^TM^ MyOne^TM^ Tosylactivated was placed on a glass slide under a coverslip with one drop (~50 µL) of SlowFade^TM^ Diamond Antifade Mountant (Molecular Probes #S36967). Beads were excited using a 532 nm laser and imaged from 554 nm to 697 nm with a Zeiss LSM 780 Confocal Microscope equipped with a 20X water immersion objective (NA 1.0, Zeiss #421452-9800-000).

Maximum intensity Z-projections of each Z-stack were generated and thresholded (binarized) using ImageJ and the Default Local Threshold algorithm. The Analyze Particles plugin from ImageJ was used to identify and measure the area of each bead.

## 3. Results and Discussion

### 3.1. Bead-Based QLISA Development

**Figure 1:**
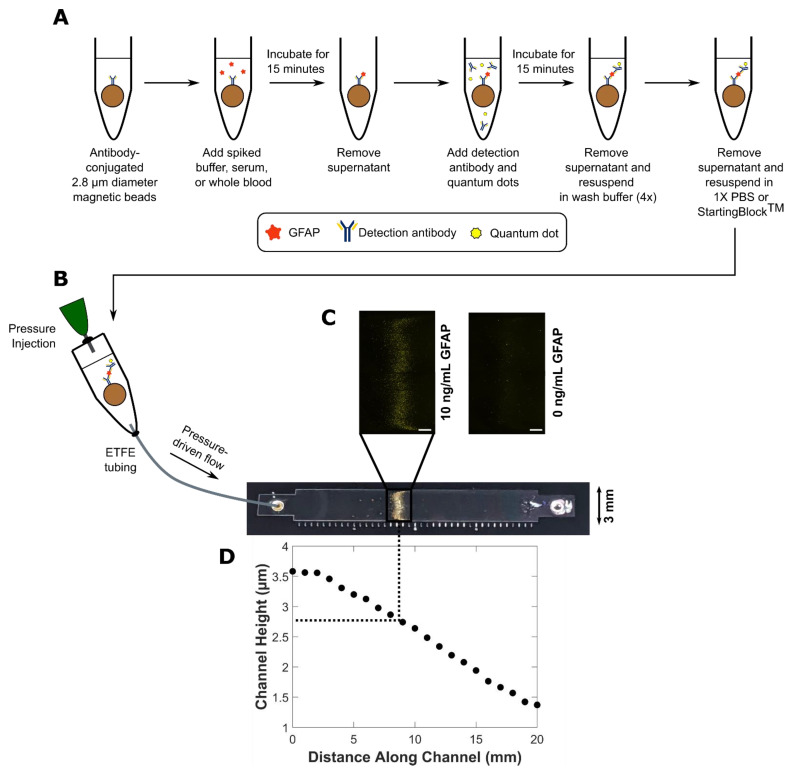
Schematic of the method used to run and analyze bead-based quantum dot-linked immunosorbent assays (QLISAs) for glial fibrillary acidic protein (GFAP) (pictured) as well as interleukin-6 (IL-6) and interleukin-8 (IL-8). (**A**) The binding steps necessary to complete the sandwich assay were performed on the benchtop in a microcentrifuge tube. Note that a single bead is pictured with a single antibody for the sake of clarity, but the assay consisted of millions of beads completely coated in antibodies. (**B**) The bead mixture with completed sandwich complexes was introduced into the variable height device using a modified microcentrifuge tube and pneumatic pressure. (**C**) The beads formed fluorescent bands inside the variable height device where the fluorescence intensity is related to the protein concentration in the sample (white scale bar 500 µm). (**D**) The channel height varies between the inlet and outlet, so the assay beads become trapped where their diameter matches the height of the channel (graph is of a representative height profile obtained using a stylus profilometer).

**Figure 2:**
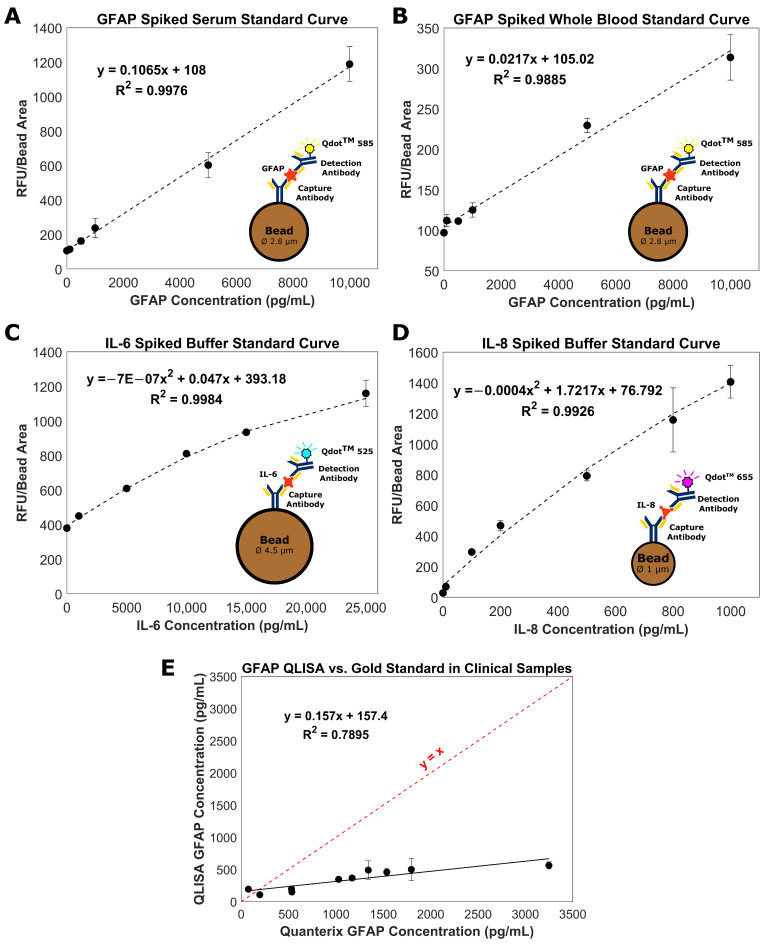
QLISA standard curves for GFAP in (**A**) spiked human serum and (**B**) spiked human whole blood, (**C**) IL-6 in spiked buffer, and (**D**) IL-8 in spiked buffer. Each data point represents three replicates (*n* = 3). (**E**) Comparison of the GFAP concentrations determined by the Quanterix Simoa^®^ assay and by the QLISA method for ten clinical serum samples. The dashed red line represents the line *y* = *x*, and the solid black line represents the trend between the Quanterix GFAP concentration and the QLISA GFAP concentration. Each black dot represents a serum sample from a different donor. Replicates ranged from n = 1 to n = 5 depending on the volume of the serum sample. All error bars throughout the figure are standard error of the mean.

**Figure 3:**
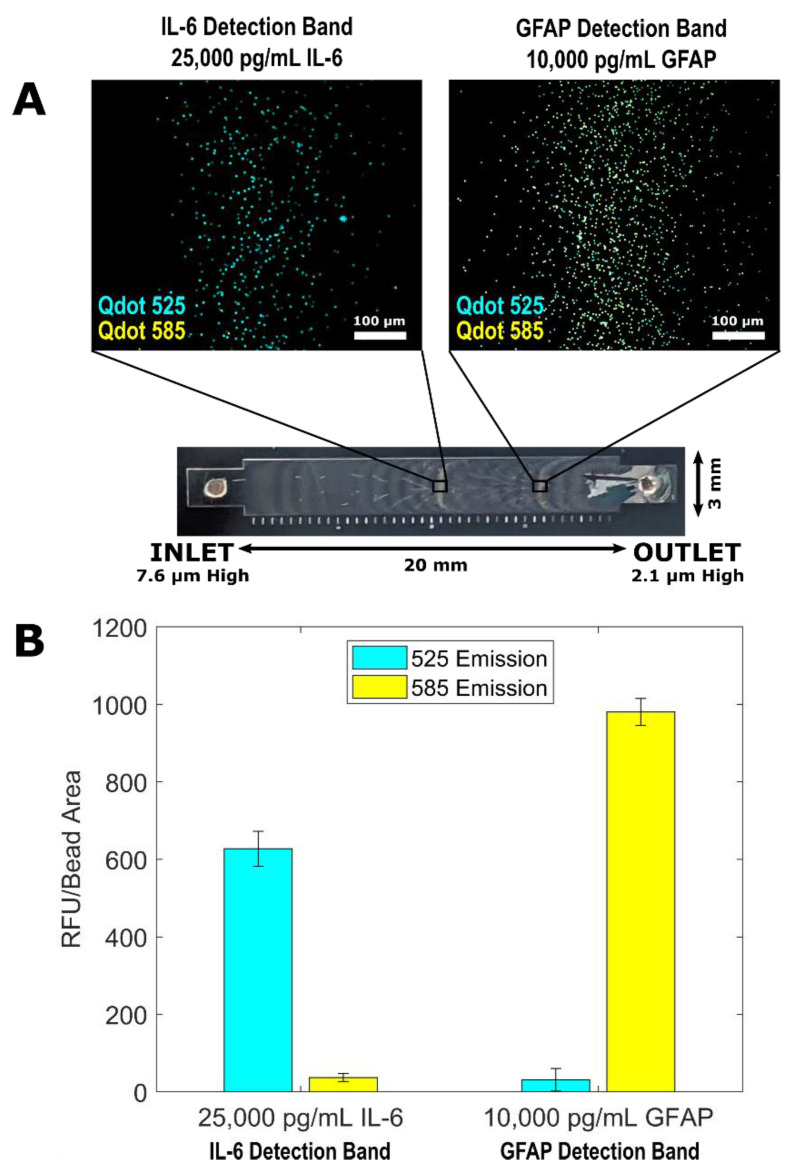
Multiplex of the IL-6 QLISA (fluorescent signal from Qdot^TM^ 525) and the GFAP QLISA (fluorescent signal from Qdot^TM^ 585) where the sample consisted of 25,000 pg/mL IL-6 and 10,000 pg/mL GFAP. (**A**) Representative images of ~4.5 µm beads trapped in the IL-6 detection band of the variable height device (left) and ~2.8 µm beads trapped in the GFAP detection band (right). Each image is an overlay of the Qdot^TM^ 525 and Qdot^TM^ 585 channels, and the brightness/contrast was set for ease of viewing. (**B**) The fluorescence intensity values from the Qdot^TM^ 525 (blue) and Qdot^TM^ 585 (yellow) channels for each variable height device detection band shown in (**A**) with the control values subtracted. The error bars are standard error of the mean and represent the variability in fluorescence intensity between the fields of view within each detection band.

**Figure 4:**
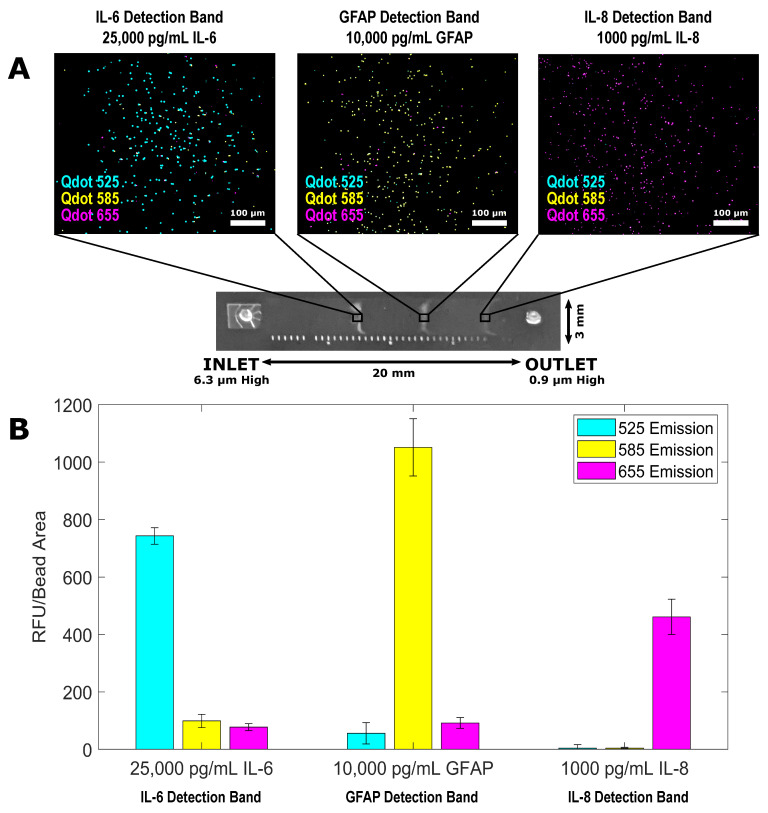
Multiplex of the IL-6 QLISA (fluorescent signal from Qdot^TM^ 525), the GFAP QLISA (fluorescent signal from Qdot^TM^ 585), and the IL-8 QLISA (fluorescent signal from Qdot^TM^ 655) where the sample consisted of 25,000 pg/mL IL-6, 10,000 pg/mL GFAP, and 1000 pg/mL IL-8. (**A**) Representative images of ~4.5 µm beads trapped in the IL-6 detection band of the variable height device (left), ~2.8 µm beads trapped in the GFAP detection band (center), and ~1 µm beads trapped in the IL-8 detection band (right). Each image is an overlay of the Qdot^TM^ 525, Qdot^TM^ 585, and Qdot^TM^ 655 channels. The brightness/contrast of each image was set for ease of viewing. (**B**) The fluorescence intensity values from the Qdot^TM^ 525 (blue), Qdot^TM^ 585 (yellow), and Qdot^TM^ 655 (magenta) channels for each variable height device detection band shown in (**A**) with the control values subtracted. The error bars are standard error of the mean and represent the variability in fluorescence intensity between the fields of view within each detection band.

**Figure 5:**
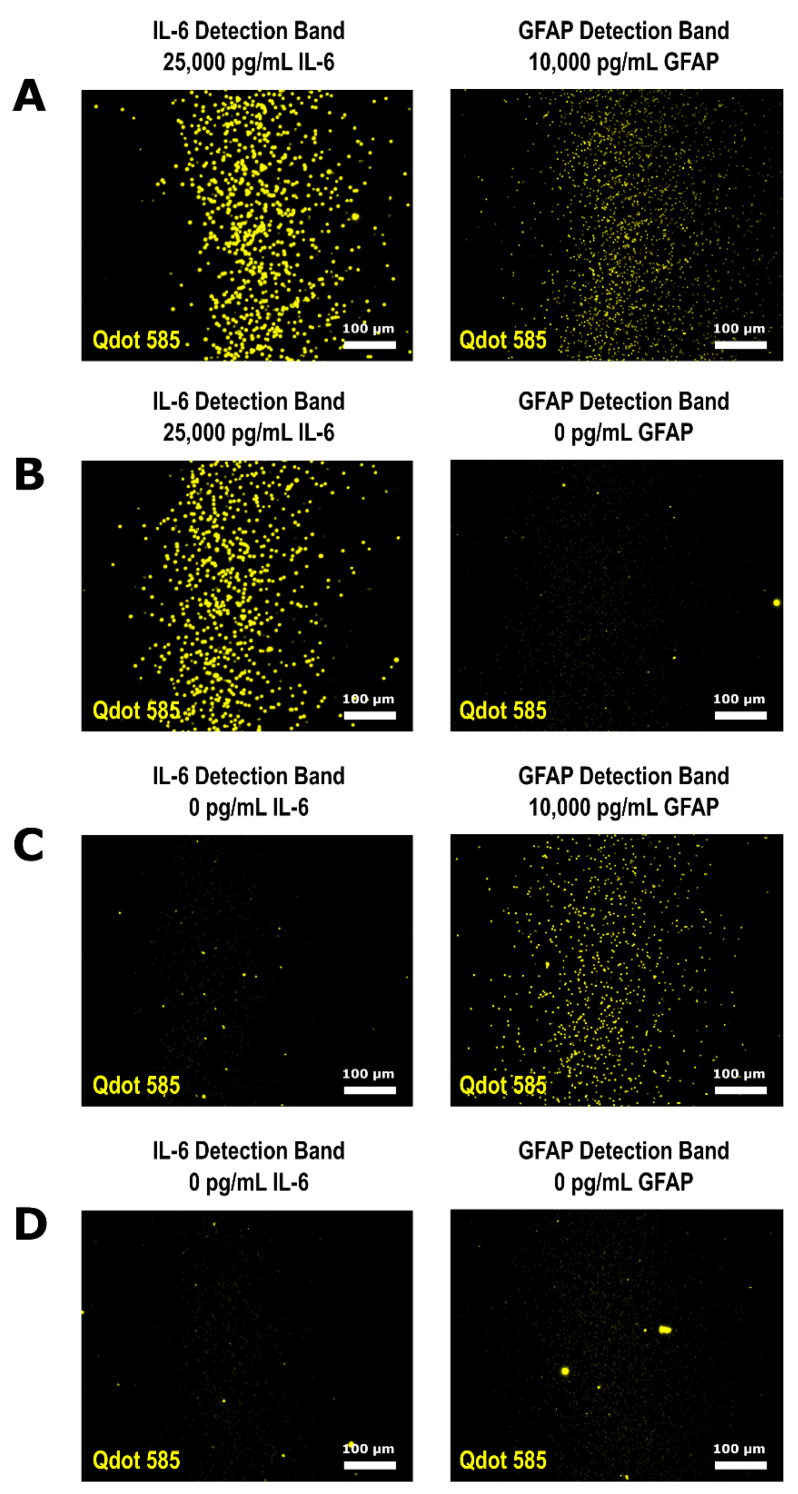
Multiplex of the IL-6 QLISA and the GFAP QLISA where the fluorescent signal for both assays is from Qdot^TM^ 585. Both assays were run in the same microcentrifuge tube on the same spiked buffer sample. The representative fluorescence images from the variable height device detection bands of (**A**) a sample containing 25,000 pg/mL IL-6 and 10,000 pg/mL GFAP, (**B**) a sample containing 25,000 pg/mL IL-6 and 0 pg/mL GFAP, (**C**) a sample containing 0 pg/mL IL-6 and 10,000 pg/mL GFAP, and (**D**) a sample containing 0 pg/mL IL-6 and 0 pg/mL GFAP all have the same brightness/contrast for ease of visual comparison.

The assay steps described in Section 2.2, Section 2.4 and Section 2.5 and depicted in Figure 1A were carried out on the benchtop. The assay beads with completed sandwich complexes were then introduced into the variable height device (Figure 1B) consisting of a single channel that varies in height between the inlet and the outlet (Figure 1D). The assay beads were trapped and formed distinct fluorescent bands (Figure 1C) where their diameter matched the height of the channel. A summary of the channel height profiles and assay beads used to generate the data shown in Figure 2, Figure 3, Figure 4 and Figure 5 can be found in Table 1. The diameter distribution and summary statistics for each Dynabead^TM^ can be found in Figure S1 and Table S1, respectively.

Images of the fluorescent bands were analyzed as described in Section 2.9 to form the standard curves in Figure 2. For each curve, the data points represent three replicates of a given protein concentration, and the error bars are the standard error of the mean. The LOD of the GFAP assay in serum (Figure 2A) was 125 pg/mL, the intra-assay CV (at 10,000 pg/mL) was 6%, and the inter-assay CV (at 10,000 pg/mL) was 16%. For comparison, Rickard et al. measured GFAP in human plasma using surface-enhanced Raman spectroscopy (SERS) and achieved a LOD of 3.35 pg/mL [26]. It is difficult to make direct comparisons between these methods as the sample matrices differ (serum vs. plasma). However, a LOD of 125 pg/mL offers clinically relevant sensitivity, specificity, positive predictive value, and negative predictive value when used to predict intracranial abnormalities on head CT after an mTBI [27]. For the GFAP assay in whole blood (Figure 2B), the LOD was 1112 pg/mL, the intra-assay CV (at 10,000 pg/mL) was 3%, and the inter-assay CV (at 10,000 pg/mL) was 23%. To the best of our knowledge, this is the first reported instance of measuring GFAP in spiked human whole blood.

The dynamic range of both the serum and whole blood curves was within the physiologically relevant range of GFAP concentrations post-TBI, 153–2089 pg/mL [6]. The GFAP concentration in healthy controls is 14–25 pg/mL [9]. Since the LOD of the GFAP QLISA fell between the concentration ranges indicative of TBI and healthy individuals, our assay could be capable of distinguishing patients with and without a TBI. There is a compromise on the limit of detection when using the GFAP QLISA with whole blood due to the increased complexity of the sample matrix. Modifications to the assay would need to be made to decrease the LOD in whole blood before the assay could be used to assess clinical samples.

The LOD of the IL-6 assay in buffer (Figure 2C) was 437 pg/mL, the intra-assay CV (at 10,000 pg/mL) was 5%, and the inter-assay CV (at 10,000 pg/mL) was 5%. For comparison, Messina et al. achieved a LOD of 1.56 pg/mL using a microfluidic immunosensor [28]. The LOD of the IL-8 assay in buffer (Figure 2D) was 2 pg/mL, the intra-assay CV (at 1000 pg/mL) was 13%, and the inter-assay CV (at 1000 pg/mL) was 14%. For context, Zhao et al. achieved a LOD of 23 pg/mL using an ELISA and 0.16 pg/mL using a photoacoustic immunoassay [29]. The dynamic range of both the IL-6 and IL-8 curves was within the physiologically relevant range of IL-6 and IL-8 concentrations in serum and cerebrospinal fluid (CSF) post-TBI (4–35,500 pg/mL for IL-6 and 30–8000 pg/mL for IL-8) [19]. The physiological concentrations of IL-6 and IL-8 in serum are approximately 4 pg/mL and 30 pg/mL, respectively [28,29]. Therefore, the LOD of the IL-6 assay must be improved before it can be used clinically.

We successfully developed three different QLISAs for three different TBI protein biomarkers using three different bead sizes and three different quantum dot colors. However, the system is not limited to the analytes, bead sizes, and quantum dots described above. If the chosen assay beads have surface epoxy or tosyl groups, the antibody pair is specific to the analyte of interest, and the appropriate microscope filters are available to analyze the chosen quantum dots, the design parameters discussed in Section 2.1 can be used to develop QLISAs for any analyte of interest, either for TBI or another condition of concern to human health. A current limitation of the QLISA system is an elevated LOD in comparison with other systems reported in the literature. However, the LODs of the QLISAs in this work are not optimized. Reagent or other experimental condition adjustments could bring the sensitivity in line with other published devices.

### 3.2. Measurement of GFAP in Clinical Serum Samples

The GFAP standard curve (Figure 2A) was used to determine the GFAP concentration in ten clinical serum samples from ten different donors. These clinical samples had previously been tested for GFAP using the Quanterix Simoa^®^ assay [30,31], one of the methods for measuring GFAP as part of TBI clinical trials. As seen in Figure 2E, the GFAP QLISA underestimated the GFAP concentration measured by Quanterix. It is unlikely that this discrepancy is due to an interfering substance in the serum matrix as there is a consistent trend in the GFAP QLISA values even with each serum sample coming from a different donor. 

We hypothesize that the discrepancy between the concentrations from the GFAP QLISA and Quanterix Simoa^®^ (Dortmund, Germany) assay is due to differences in the form of GFAP recovered by the antibodies. The Abcam antibodies (ab222279) used in the GFAP QLISA detect native GFAP (50 kDa). However, the Quanterix Simoa^®^ assay uses antibodies from Banyan Biomarkers^®^ that detect both native GFAP as well as GFAP breakdown products (GFAP-BDP) (50–38 kDa) [32]. GFAP is highly vulnerable to calpain-mediated proteolysis in vivo, so the form of GFAP in biofluids, such as serum, is likely to be GFAP-BDP [33,34]. Therefore, one would expect the GFAP concentration measured by the Quanterix Simoa^®^ assay to be higher than the GFAP QLISA, as seen in Figure 2E, since the Quanterix assay can detect both native GFAP and GFAP-BDP as opposed to the GFAP QLISA that can only detect native GFAP.

An outstanding question in TBI biomarker analysis is whether there are advantages to monitoring intact GFAP vs. GFAP-BDPs. It remains to be determined if intact GFAP or GFAP-BDPs better reflect the extent or kinetics of astrocyte injury following a TBI [34]. Since the GFAP QLISA can be used to quantify native GFAP, the variable height device system could potentially be used to address this question.

### 3.3. Multiplexed QLISA Analysis Using the Variable Height Microfluidic Device

The design of the variable height device lends itself to multiplexing of bead-based immunoassays. Variable height channels with a height of ~7.6 µm at the inlet and ~2.1 µm at the outlet were used to multiplex the IL-6 QLISA and GFAP QLISA (Figure 3). A mixture of Dynabeads^TM^ M-450 (~4.5 µm diameter) and Dynabeads^TM^ M-270 (~2.8 µm) with completed sandwich complexes for IL-6 and GFAP, respectively, were introduced into the channel. The beads flowed through the channel and passively separated into two distinct bands where the diameter of the beads matched the height of the channel. Because the ~2.8 µm beads flowed through the band of ~4.5 µm beads as it was forming, it is possible that the ~2.8 µm assay beads could be trapped upstream of where the channel height matched their diameter.

To visualize this potential crossover between detection bands, we used Qdot^TM^ 525 as the fluorescent signal for the IL-6 QLISA and Qdot^TM^ 585 as the fluorescent signal for the GFAP QLISA. Figure 3A displays representative fluorescence images from an IL-6 detection band (left) and a GFAP detection band (right) where the sample contained 25,000 pg/mL IL-6 and 10,000 pg/mL GFAP. Each image is an overlay of the Qdot^TM^ 525 and Qdot^TM^ 585 channels. Ideally, the IL-6 detection band should appear completely blue, and the GFAP detection band should appear completely yellow. Figure 3B shows the fluorescence intensity of the detection bands pictured in Figure 3A with the controls subtracted. As shown in the left pair of bars in Figure 3B, a few ~2.8 µm assay beads did become trapped in the IL-6 detection band. However, the fluorescence intensity from the Qdot^TM^ 585 channel (yellow bar) was minimal in comparison with the intensity from the Qdot^TM^ 525 channel (blue bar) such that it would not influence the overall protein concentration determination. In the GFAP detection band (right pair of bars in Figure 3B), the signal from the Qdot^TM^ 525 channel was due to strong autofluorescence of the ~2.8 µm assay beads at 525 nm rather than contamination by unbound quantum dots from the IL-6 detection band [35]. It is physically impossible for ~4.5 µm diameter assay beads to become trapped in the GFAP detection band due to the channel height gradient.

Following the successful multiplex of the IL-6 and GFAP QLISAs, all three QLISAs (for IL-6, GFAP, and IL-8) were multiplexed using variable height channels with a height of ~6.3 µm at the inlet and ~0.9 µm at the outlet (Figure 4). We used Qdot^TM^ 525 as the fluorescent signal for the IL-6 QLISA (~4.5 µm assay beads), Qdot^TM^ 585 as the fluorescent signal for the GFAP QLISA (~2.8 µm assay beads), and Qdot^TM^ 655 as the fluorescent signal for the IL-8 QLISA (~1 µm diameter assay beads). A mixture of the different-sized assay beads with completed sandwich complexes for the proteins of interest was introduced into the channel and allowed to passively separate. Figure 4A displays representative fluorescence images from an IL-6 detection band (left), a GFAP detection band (center), and an IL-8 detection band where the sample contained 25,000 pg/mL IL-6, 10,000 pg/mL GFAP, and 1000 pg/mL IL-8. Each image is an overlay of the Qdot^TM^ 525, Qdot^TM^ 585, and Qdot^TM^ 655 channels. Ideally, the IL-6 detection band should appear completely blue, the GFAP detection band should appear completely yellow, and the IL-6 detection band should appear completely magenta. Figure 4B shows the fluorescence intensity of the detection bands pictured in Figure 4A with the controls subtracted. As shown in the left pair of bars in Figure 4B, ~2.8 µm and ~1 µm assay beads did become trapped in the IL-6 detection band. However, the fluorescence intensity from the Qdot^TM^ 585 channel (yellow bar) and the Qdot^TM^ 655 channel (magenta bar) was minimal in comparison with the intensity from the Qdot^TM^ 525 channel (blue bar). This was similarly the case in the GFAP detection band (center pair of bars in Figure 4B), where the signal from the Qdot^TM^ 585 channel (yellow bar) was the strongest, and in the IL-8 detection band (right pair of bars in Figure 4B), where the signal from the Qdot^TM^ 655 channel (magenta bar) was the strongest. As in the two-quantum dot color multiplex of IL-6 and GFAP, the signal from the Qdot^TM^ 525 channel (blue bar) in the GFAP detection band was due to strong autofluorescence of the assay beads at 525 nm.

There was minimal crossover between the detection bands when the two-plex and three-plex were run using different Qdot^TM^ colors. Therefore, we performed a multiplex using Qdot^TM^ 585 as the fluorescent signal for both the IL-6 QLISA and GFAP QLISA by combining all assay reagents in the same microcentrifuge tube. The results of this single microcentrifuge tube multiplex are presented in Figure 5, where each part (A–D) represents a single variable height channel with an IL-6 detection band and a GFAP detection band. Using only one quantum dot color, we quantified IL-6 and GFAP in samples containing 25,000 pg/mL IL-6 and 10,000 pg/mL GFAP (Figure 5A), 25,000 pg/mL IL-6 and 0 pg/mL GFAP (Figure 5B), 0 pg/mL IL-6 and 10,000 pg/mL GFAP (Figure 5C), and 0 pg/mL IL-6 and 0 pg/mL GFAP (Figure 5D). As expected from the multiplex using two quantum dot colors (Figure 3), a few ~2.8 µm assay beads were trapped in the IL-6 detection band (Figure 5C).

To investigate the potential of the bead crossover between detection bands to generate false positive signals, we statistically compared the fluorescent intensity of the IL-6 detection bands in Figure 5C (0 pg/mL IL-6 run simultaneously with 10,000 pg/mL GFAP) and Figure 5D (0 pg/mL IL-6 run simultaneously with 0 pg/mL GFAP). The fluorescent intensity of the IL-6 detection bands in Figure 5C,D was not found to be significantly different when compared using a two-tailed *t*-test (*p* = 0.42), indicating that the co-trapped beads would not influence the protein concentration determination. Additionally, the bright spots in the GFAP detection band in Figure 5B and both the IL-6 and GFAP detection bands in Figure 5D are due to debris and aggregated quantum dots that are not tethered to a specific bead [36,37]. The image analysis method described in Section 2.9 minimizes the impact of debris and quantum dot aggregates as the comparison between the epi-illuminated image and fluorescence image is designed to eliminate fluorescent signal that is not directly tethered to a bead. If further experimentation and device development does lead to false positive signals due to bead crossover, a low abundance target could be run as a downstream detection band to minimize the potential for bead crossover effects or in a parallel channel on the same chip.

We successfully performed a two-plex and three-plex using different Qdot^TM^ colors as well as a two-plex using the same Qdot^TM^ color for both assays. During the two-plex assays, the mixture of assay beads separated into distinct assay bands after 4 min, but during the three-plex assay, the mixture of beads took 40 min to separate. To shorten the time it takes to form detection bands, care should be used when selecting the bead size associated with each QLISA. The bead diameter determines the height profile of the channel, and the height profile in turn determines the flow rate according to Equation S15 in the Supplementary Materials. As seen in Figures S3 and S4, the flow rate in the variable height device is strongly influenced by the channel outlet height. For a fixed outlet height, increasing the channel inlet height does little to increase the flow rate through the channel. Therefore, it is recommended that the channel outlet height should be greater than or equal to 1.5 µm to ensure that detection bands form within 15 min or less and that the assay beads do not aggregate or adsorb onto the channel walls.

## 4. Conclusions

Here was presented a variable height microfluidic device that is capable of passively multiplexing bead-based QLISAs for IL-6, GFAP, and IL-8, three TBI protein biomarkers. The assay design principles presented can be applied to any analyte of interest for which a sandwich or competitive immunoassay is feasible, making the QLISA method widely applicable to diagnosing and monitoring conditions that impact human health. Our platform decouples the QLISAs from the variable height channel, allowing for highly flexible multiplexing. Any mixture of antibody-conjugated beads of different diameters can be introduced into the channel, and the beads will passively separate into distinct bands as they flow through the channel, resulting in a custom barcode of biomarker concentrations. There is no need to actively pattern antibodies in the channel during fabrication, as the differences in bead size coupled with the channel height gradient lead to passive patterning. We used the variable height device to successfully multiplex QLISAs for IL-6 (~4.5 µm diameter assay beads) and GFAP (~2.8 µm diameter assay beads) as well as QLISAs for IL-6, GFAP, and IL-8 (~1 µm diameter assay beads). However, additional biomarkers could be multiplexed simply by adding in antibody-conjugated beads of a different diameter.

Ideally, the variable height device can be used to multiplex up to 20 different biomarkers. Each band of trapped beads is approximately 500 µm wide, and a channel height profile slope should be chosen that results in at least a 500 µm separation between the detection bands. Therefore, a 20 mm long channel could accommodate 20 different bands of beads, effectively multiplexing 20 different biomarkers. It is imperative to select assay beads that are homogeneous in size to facilitate the multiplex. Bead sizes should also be selected such that the channel outlet height is 1.5 µm or greater to ensure that the flow rate in the channel is sufficient to separate the beads within 15 min and prevent bead aggregation and adsorption. As more bead sizes are added to the channel, bead crossover between detection bands will increase. Each band of beads acts as a leaky filter as it forms, trapping some smaller diameter assay beads while allowing the majority to pass through. Detection band crossover can be mitigated by optimizing the number of beads of each size introduced into the channel such that fewer larger diameter beads are introduced, delaying the formation of upstream detection bands while downstream bands form. Of course, multiplexing of biomarkers in the variable height device is limited by antibody specificity and affinity for an analyte of interest. If an antibody is cross-reactive, multiplexing will be limited.

Since the assay beads spatially separate into distinct bands due to the ramp-like height profile of the channel, the same quantum dot can be used to detect each biomarker, simplifying the development of a point-of-care optics module. In this work, a fluorescence microscope was used to analyze the fluorescence intensity of the bands of beads, but a portable optics module would allow our device to be used wherever it is needed. A portable platform could be used by military personnel for rapid triage in the field, by coaches to assess injured players on the sidelines, by paramedics to assess a patient’s condition before arrival at a hospital, by clinicians to monitor a patient’s response to neuroprotective treatments, and by clinical research teams to accurately enroll and group patients for clinical trials. Ultimately, our platform has the potential to not only transform the field of TBI diagnosis but to expand along with it.

## Figures and Tables

**Table 1:** Variable height channel parameters and assay beads used to generate the data in Figure 2, Figure 3, Figure 4 and Figure 5. All channels had the same general height profile shown in Figure 1D between the inlet and outlet heights listed.

Figure	Inlet Height (µm)	Outlet Height (µm)	Assay Beads
2A	3.675 ± 0.081	1.386 ± 0.053	Dynabeads^TM^ M-270
2B	4.066 ± 0.006	1.313 ± 0.041	Dynabeads^TM^ M-270
2C	7.008 ± 0.278	1.848 ± 0.020	Dynabeads^TM^ M-450
2D	3.157 ± 0.045	0.856 ± 0.035	Dynabeads^TM^ MyOneTM
2E	3.653 ± 0.044	1.368 ± 0.018	Dynabeads^TM^ M-270
3A	7.583 ± 0.177	2.077 ± 0.007	Dynabeads^TM^ M-450 and M-270
4A	6.342 ± 0.035	0.963 ± 0.057	Dynabeads^TM^ M-450, M-270, and MyOne^TM^
5A–E	7.583 ± 0.177	2.077 ± 0.007	Dynabeads^TM^ M-450 and M-270

Note: All channels were 3 mm wide and 2 cm long.

## Data Availability

Not applicable.

## Supplementary Materials

The following are available online at https://www.mdpi.com/article/10.3390/bios11090320/s1: Figures S1–S4, Table S1, and Equations (S1)–(S15).
