# Supplementary material for: A Variable Height Microfluidic Device for Multiplexed Immunoassay Analysis of Traumatic Brain Injury Biomarkers
Source: Biosensors (Basel). 2021 Sep 7;11(9):320. doi: 10.3390/bios11090320 (PMC8472232; doi:10.3390/bios11090320)
Supplement: Supplementary file 1 [file biosensors-11-00320-s001.zip › biosensors-1352216-supplementary.pdf]

Supplementary Materials

S1. Bead size characterization

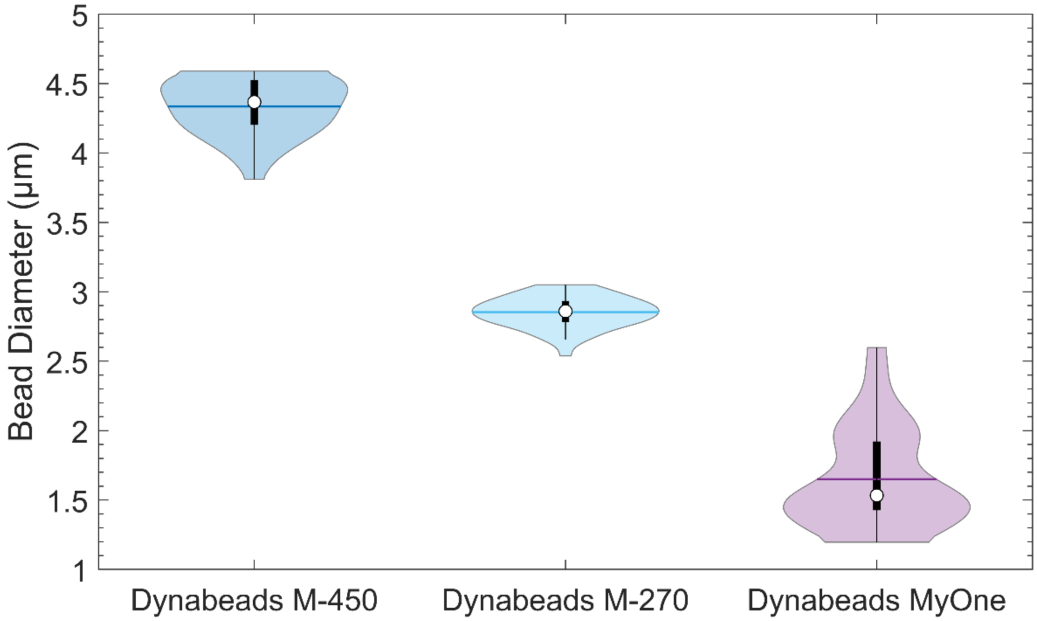

Figure S1. Violin plots showing the distribution of bead diameters for different types of Dynabeads™ from Invitrogen™. These distributions were obtained by imaging the beads using a confocal microscope at 20X magnification and analyzing the thresholded images using the Analyze Particles plugin from ImageJ.

Table S1: Dynabead™ diameter statistics.

Bead Name	Reported Mean (μm)	Measured Mean (μm)	Measured S.D. (μm)
Dynabeads™ M-450 Epoxy	4.5	4.34	0.20
Dynabeads™ M-270 Epoxy	2.8	2.85	0.11
Dynabeads™ MyOne™ Tosylactivated	1.0	1.65	0.36

S2. Derivation of the flow rate equation for variable height channels

Pressure-driven flow in a rectangular channel of constant height can be described according to:

$$Q = \frac{wh^3 \Delta p}{12\mu L} \tag{S1}$$

where w is the channel width, h is the channel height, μ is the dynamic viscosity of the fluid, and Δp is the pressure drop along the channel length, L. (See Stone, H. A. (2007). Introduction to fluid dynamics for microfluidic flows. In *CMOS Biotechnology* (pp. 5-30). Springer, Boston, MA.)

The height, pressure, and length parameters of a variable height channel are illustrated in Figure S2.

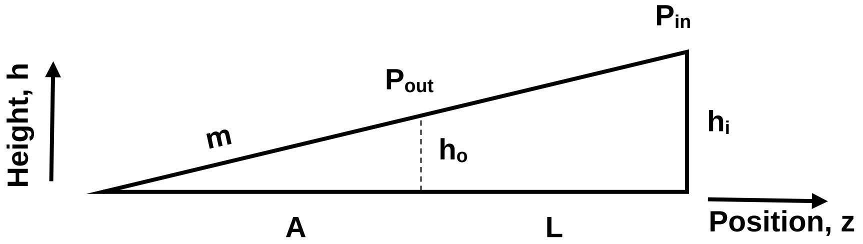

Figure S2. The height profile of a variable height device can be modeled as a triangle where h_i is the inlet height, h_o is the outlet height, P_{in} is the pressure at the inlet, P_{out} is the pressure at the outlet, m is the height profile slope, and A and L describe lengths of the channel.

From Figure S2, the following equations emerge:

$$m = \frac{h_i - h_o}{L} \tag{S2}$$

$$m = \frac{h_o}{A} \tag{S3}$$

$$A = \frac{L h_o}{h_i - h_o} \tag{S4}$$

$$A = \frac{h_o}{m} \tag{S5}$$

$$h = mz \; from \; z = A \; to \; z = A + L \tag{S6}$$

Rearranging Equation S1, we arrive at:

$$\frac{dP}{dz} = \frac{\Delta p}{L} = \frac{12\mu Q}{wh^3} \tag{S7}$$

Substituting Equation S6 in for h gives us:

$$dP = \frac{12\mu Q}{wm^3 z^3} dz \tag{S8}$$

which we can integrate over the length of the channel:

$$\int_{P_{out}}^{P_{in}} dP = \int_{A}^{A+L} \frac{12\mu Q}{wm^3 z^3}\, dz \tag{S9}$$

to yield:

$$P_{in} - P_{out} = \frac{-6\mu Q}{wm^3}\left[\frac{1}{(A+L)^2} - \frac{1}{A^2}\right] \tag{S10}$$

In this case, the outlet of the variable height device is open to the atmosphere, so $P_{out} = P_{atm} = 0$. Therefore, Equation S10 becomes:

$$P_{in} = \frac{-6\mu Q}{wm^3}\left[\frac{1}{(A+L)^2} - \frac{1}{A^2}\right] \tag{S11}$$

Simplifying Equation S11 results in:

$$P_{in} = \frac{12\mu QL}{w(mA)^3}\left[\frac{1 + \frac{L}{2A}}{(1 + \frac{L}{A})^2}\right] \tag{S12}$$

Substituting in h_o for mA (Equations S3 and S5) and Equation S4 for A yields:

$$P_{in} = \frac{12\mu QL}{w{h_o}^3}\left[\frac{1 + \frac{h_i - h_o}{2h_o}}{(1 + \frac{h_i - h_o}{h_o})^2}\right] \tag{S13}$$

Simplifying further results in:

$$P_{in} = \frac{6\mu QL}{w{h_o}^2{h_i}^2}(h_o + h_i) \tag{S14}$$

which we can rearrange for Q:

$$Q = \frac{w{h_o}^2{h_i}^2P_{in}}{6\mu L(h_o + h_i)} \quad \text{(S15)}$$

It is worth noting that when $h_o = h_{in} = h$, Equation S15 becomes Equation S1.

For all the data presented in this manuscript, $\frac{wP_{in}}{\mu L}$ was constant. Therefore, Q can be plotted as a function of h_i and h_o.

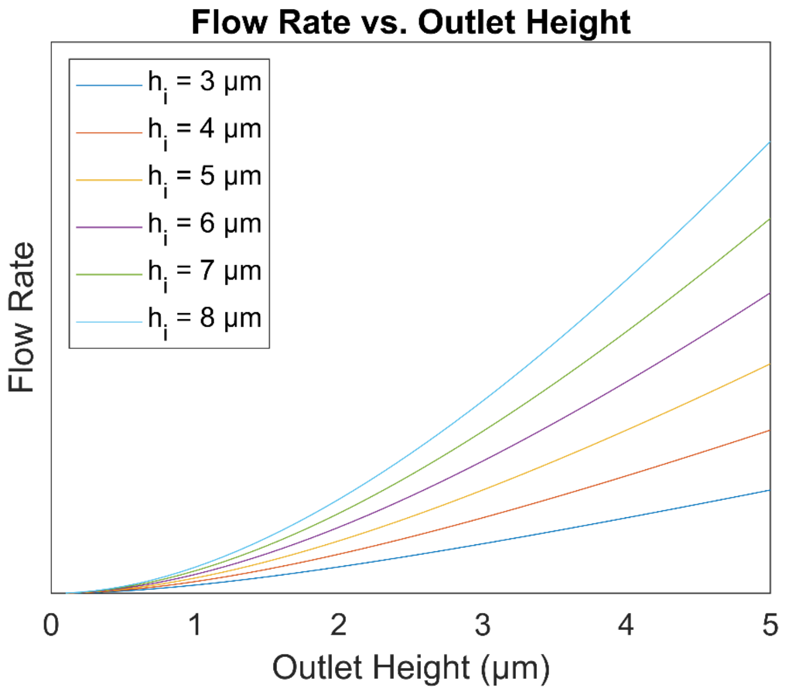

Figure S3: Relationship between flow rate and outlet height generated using Equation S15. Numbers for flow rate are not provided as it varies with inlet pressure, and exact flow rates can be calculated using Equation S15.

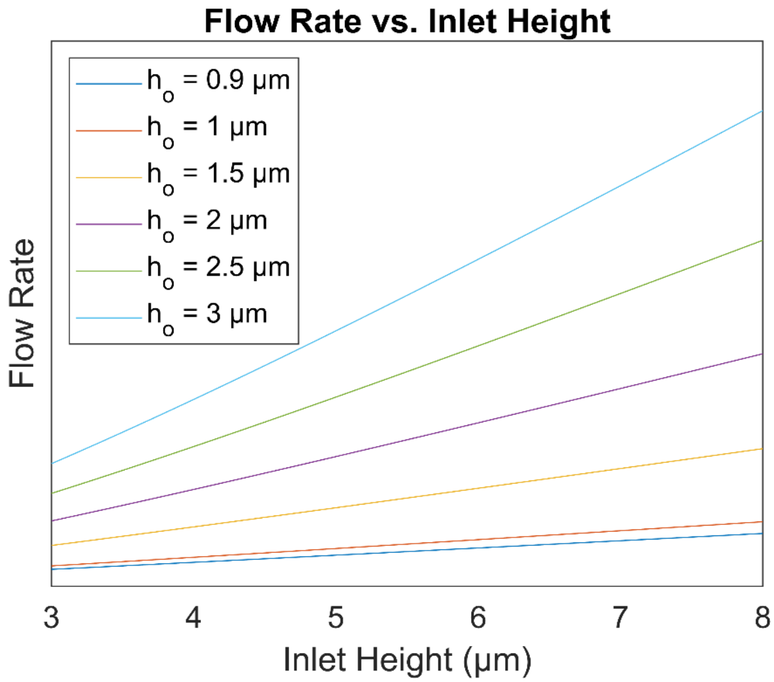

Figure S4: Relationship between flow rate and inlet height generated using Equation S15. Numbers for flow rate are not provided as it varies with inlet pressure, and exact flow rates can be calculated using Equation S15.
